# Mendelian randomisation analyses find pulmonary factors mediate the effect of height on coronary artery disease

**DOI:** 10.1038/s42003-019-0361-2

**Published:** 2019-03-27

**Authors:** Eirini Marouli, M. Fabiola Del Greco, Christina M. Astley, Jian Yang, Shafqat Ahmad, Sonja I. Berndt, Mark J. Caulfield, Evangelos Evangelou, Barbara McKnight, Carolina Medina-Gomez, Jana V. van Vliet-Ostaptchouk, Helen R. Warren, Zhihong Zhu, Joel N. Hirschhorn, Ruth J. F. Loos, Zoltan Kutalik, Panos Deloukas

**Affiliations:** 1 William Harvey Research Institute, Barts and The London School of Medicine and Dentistry, Queen Mary University of London, London, EC1M 6BQ UK; 2 Centre for Genomic Health, Life Sciences, Queen Mary University of London, London, EC1M 6BQ UK; 3 Institute for Biomedicine, Eurac Research, Affiliated Institute of the University of Lubeck, Bolzano, 39100 Italy; 4 Boston Children’s Hospital, Boston, MA 02115 USA; 5 Broad Institute of Harvard and MIT, Cambridge, MA 02142 USA; 6 Institute for Molecular Bioscience, University of Queensland, Brisbane, 4072 QLD Australia; 7 Queensland Brain Institute, The University of Queensland, Brisbane, 4072 QLD Australia; 8 Department of Nutrition, Harvard T.H. Chan School of Public Health, Harvard University, Boston, MA 02115 USA; 9 Division of Preventive Medicine, Harvard Medical School, Department of Medicine, Brigham and Women’s Hospital, Boston, MA 02215 USA; 10 Department of Medical Sciences, Molecular Epidemiology, Uppsala University, Uppsala, 751 41 Sweden; 11 Division of Cancer Epidemiology and Genetics, National Cancer Institute, National Institutes of Health, Department of Health and Human Services, Bethesda, MD 20892 USA; 12 National Institute for Health Research, Barts Cardiovascular Biomedical Research Center, Queen Mary University of London, London, EC1M 6BQ UK; 13 Department of Epidemiology and Biostatistics, School of Public Health, Imperial College London, London, W2 1PG UK; 14 Department of Hygiene and Epidemiology, University of Ioannina Medical School, Ioannina, 45110 Greece; 15 Department of Biostatistics, University of Washington, Seattle, WA 98101 USA; 16 Department of Internal Medicine, Erasmus Medical Center, Rotterdam, 3015 GE The Netherlands; 17 Department of Epidemiology, Erasmus Medical Center, Rotterdam, 3015 GE The Netherlands; 18 Department of Endocrinology, University of Groningen, University Medical Center Groningen, Groningen, 9713 GZ The Netherlands; 19 The Charles Bronfman Institute for Personalized Medicine, Icahn School of Medicine at Mount Sinai, New York, NY 10029 USA; 20 Institute of Social and Preventive Medicine, Lausanne University Hospital, Lausanne, 1010 Switzerland; 21 Swiss Institute of Bioinformatics, Lausanne, 1015 Switzerland; 22 Princess Al-Jawhara Al-Brahim Centre of Excellence in Research of Hereditary Disorders (PACER-HD), King Abdulaziz University, Jeddah, 21589 Saudi Arabia

## Abstract

There is evidence that lower height is associated with a higher risk of coronary artery disease (CAD) and increased risk of type 2 diabetes (T2D). It is not clear though whether these associations are causal, direct or mediated by other factors. Here we show that one standard deviation higher genetically determined height (~6.5 cm) is causally associated with a 16% decrease in CAD risk (OR = 0.84, 95% CI 0.80–0.87). This causal association remains after performing sensitivity analyses relaxing pleiotropy assumptions. The causal effect of height on CAD risk is reduced by 1–3% after adjustment for potential mediators (lipids, blood pressure, glycaemic traits, body mass index, socio-economic status). In contrast, our data suggest that lung function (measured by forced expiratory volume [FEV1] and forced vital capacity [FVC]) is a mediator of the effect of height on CAD. We observe no direct causal effect of height on the risk of T2D.

## Abstract (web summary)

Eirini Marouli et al. use Mendelian randomisation analyses to investigate the causal relationship between adult height, coronary artery disease (CAD) and type 2 diabetes (T2D) in the UK Biobank. They find that height has a causal effect on CAD, which is mediated by lung function, while there is no direct effect on the risk of T2D.

## Introduction

Evidence from observational studies suggests that height is associated with different disease outcomes^1–4^. Other studies have tried to elucidate this inverse association by using a twin design^5^ or Mendelian randomisation approaches^4,6,7^. A decrease of one standard deviation in genetically determined height (~6.5 cm) has been associated with a 13% higher risk of coronary artery disease (CAD)^4^. Health is sometimes compromised in favour of immediate survival or reproduction^8^. Subtle trade-offs are both predicted and observed in growth, health and reproduction^9^. Another trade-off is between reproduction and longevity, with many studies indicating that parental survival declined in proportion to the number of children produced^10^. There is also controversial evidence suggesting taller populations are not always in lower risk of CAD^11^.

In situations where randomised trials are inappropriate or impossible, Mendelian randomisation provides a good alternative to study the causal relationship between a trait and a disease outcome. Mendelian randomisation which is an instrumental variable-based method to infer causality in observational studies^12^, can therefore be applied to investigate any causal effect that adult height may have on cardiometabolic outcomes, and provide some insight about potential mechanisms. Mendelian randomisation offers major advantages; for example, germ-line genetic variants are assorted during formation of gametes prior to conception and are not confounded by lifestyle or environmental factors in ethnically homogeneous samples of unrelated individuals. Thus, it becomes possible to investigate how height variants may affect cardiometabolic risk and whether this effect is direct or mediated through other biological pathways.

Mendelian randomisation relies on the availability of genetic variants robustly associated with height. So far, large-scale meta-analyses of genome-wide association studies (GWAS) have identified circa 600 loci associated with adult height^13,14^ harbouring over 960 independent associations. In our latest study, height-increasing alleles at all 606 height-associated variants (Exome Chip data) were enriched for nominally significant protective effect on several cardiometabolic traits: total cholesterol (TC; *P*binomial = 4.4 × 10^−8^), triglyceride (TG; *P*binomial = 8.9 × 10^−7^) and coronary artery disease (CAD; *P*binomial = 6.0 × 10^−10^).

Besides CAD, greater adult stature has also been reported to be associated with lower risk of type 2 diabetes (T2D)^15^. Adult stature is the result of bone elongation. Bone serves as a scaffold for other organs and is an endocrine organ involved in the regulation of glucose and energy metabolism. Consequently, hormones implicated in bone remodelling may affect risk of cardiometabolic disease^16^. Adult height has also been associated with cardiorespiratory mortality^17^. Epidemiological studies have reported that much of this association can be attributed to lung function^17^ and there is evidence suggesting that measures of lung development can serve as biomarkers for childhood exposures that may modify an individual’s risk of developing CAD^18^. Previous efforts have reported that taller individuals have a lower risk of CAD giving as potential explanations that taller people have a better lung function^7^, but it is still unclear whether and to which extent lung function mediates this effect.

In general, height has an important partial role in determining several aspects of an individual’s socioeconomic status, including education, income and job class^19^. Height has been also reported to have a strong positive effect on educational attainment, with 2.5 additional centimetres in height yielding one additional month of schooling^20^. There is also support for low education as a causal risk factor in the development of CAD^21^. Previous studies have tried to clarify the associations between height and its association with CAD risk factors^4,6,7^, but it is still unclear which risk factors, mediate the inverse association between height and CAD.

Here, we test whether height is causally related to cardiometabolic disease (CAD and T2D), including traditional risk factors in the first instance. We undertake Mendelian randomisation analyses in UK Biobank (UKBB)^22^ by using a comprehensive set of height associated variants. We perform instrumental variable analysis using individual data and 828 of the previously established height-associated SNPs, which explain around 30% of height variation^13,14^. In this context we investigate glycaemic measures (glucose, insulin, glycated haemoglobin (HBA1c), 2 h postprandial glucose-2hGlu); blood pressure measures (systolic blood pressure (SBP), diastolic blood pressure (DBP), pulse pressure); obesity traits (body mass index, BMI); lipid measures (total cholesterol, low density lipoprotein (LDL), high density lipoprotein (HDL), triglycerides); and lung function measures (forced expiratory volume in 1 s (FEV1) and forced vital capacity (FVC)). We also take into account socio-economic status variables including: age in years at completion of full time education, education coded as college or University degree, income variable representing annual household income before tax and downsend deprivation index (a composite measure of deprivation based on unemployment, non-car ownership, non-home ownership and household overcrowding). Our results show that increased height reduces the risk of CAD by 16% and traditional risk factors attenuate this effect by only 1–3% suggesting different mediating pathways. Adjustment for the genetic effect of lung function (measured by FEV1 and FVC) completely abolishes the effect of height on CAD. We do not observe any direct effect of height on T2D risk.

## Results

### Study overview

**Fig. 1:**
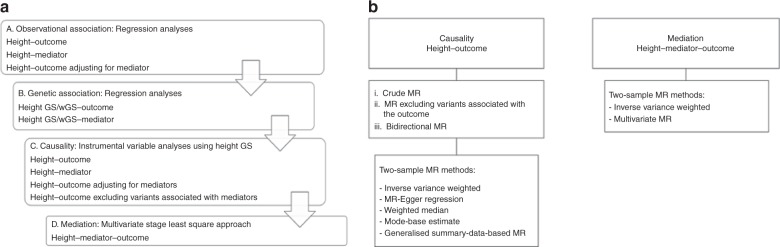
Flowchart of the study design. **a** Using individual level data from UK Biobank. **b** Using summary data

To test whether genetically determined height is related to cardiometabolic disease phenotypes, independently of traditional risk factors, we undertook Mendelian randomisation analyses in UKBB including 449,094 unrelated British participants with both phenotypic and genetic data (Supplementary Data 1). Mean height was 168.53 cm (range 125–209 cm); 23,755 individuals had CAD and 29,427 had T2D (see Methods for inclusion criteria). To perform Mendelian randomisation analyses, we constructed an unweighted and a weighted genetic score using 828 SNPs associated with adult height. The two scores were normally distributed in UKBB and robustly associated with height, as expected, in this cohort (Supplementary Figs 1–2). A flowchart of all analyses undertaken to investigate the effect of height on cardiometabolic disease risk (CAD and T2D) and possible mediators is presented in Fig. 1.

### Measured and genetically determined height associations

We initially tested for association between measured adult height and cardiometabolic diseases (CAD and T2D) (Supplementary Data 2a). A one standard deviation increase in height was associated with an odds ratio (OR) of 0.82 (95% CI 0.81–0.83) and 0.89 (95% CI 0.87–0.90) for risk of CAD and T2D, respectively, consistent with previously reported associations^3,4^. We also tested the effect of height on cardiometabolic disease by taking into account risk factors, but the observed effects were not affected (Supplementary Data 2b). A higher genetic score was associated with a protective effect on CAD risk (OR = 0.78, 95% CI 0.74, 0.82) and T2D risk (OR = 0.91, 95% CI 0.87–0.94) (Supplementary Data 3).

### Mendelian randomisation analyses

Having established observational and genetic associations between adult height and CAD and T2D risk respectively, we set to perform Mendelian randomisation analyses to further investigate whether this relationship is causal or not.

### Instrumental variable analysis in the UKBB

**Fig. 2:**
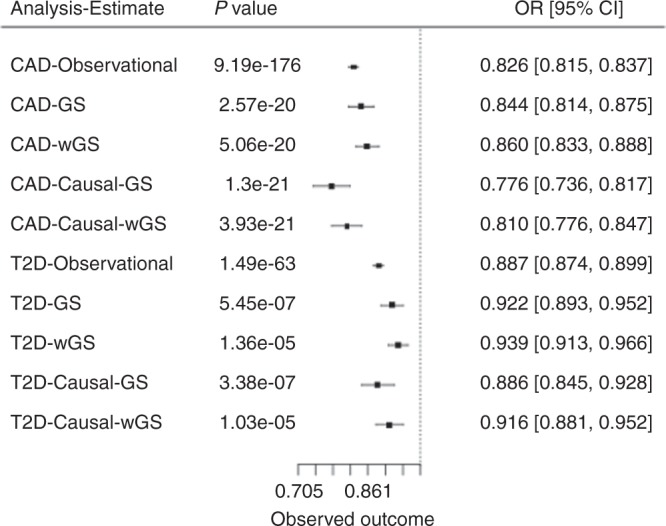
Observational and instrumental variables estimates of the effect of height on cardiometabolic events. Effect estimates represent the OR (95% CI) per 1 standard deviation increase in height, observational estimates were adjusted for age and sex. Causal estimates were derived from instrumental variable (IV) analysis

Two-stage analyses for CAD and T2D events in UKBB, using either the unweighted or the weighted genetic score (Supplementary Data 4a), showed in all instances an inverse association. For the genetic score, a 1 standard deviation higher height was associated with an OR of 0.77 (95% CI 0.73–0.81) for CAD and an OR of 0.90 (95% CI 0.86–0.94) for T2D. A similar effect was observed when using the weighted genetic score instrument (OR of 0.81 (95% CI 0.77–0.84) for CAD and an OR of 0.93 (95% CI 0.89–0.96) for T2D) (Supplementary Data 4a, Fig. 2).

For both CAD and T2D, we performed two-stage analysis adjusted for one risk factor at the time (BMI, SBP, DBP, hypercholesterolaemia) in order to estimate the causal effect for height that is independent of each cardiometabolic factor. Our results suggest that the effect of height on CAD is independent of each risk factor tested whereas its effect on T2D was completely abolished after adjustment for BMI (for genetic score: OR = 0.98, 95% CI 0.94–1.04, *p* = 0.667) (Supplementary Data 4b). Sex stratified analyses did not affect the results for CAD, but the evidence of causality of height on T2D was attenuated in females when using weighted genetic score as instrument. For CAD, the magnitude of the effect and the significance level were lower in females compared to males (females: OR = 0.82, 95% CI 0.73–0.92, *p* = 3.16 × 10^−4^ and males: OR = 0.77, 95% CI 0.73–0.82, *p* = 1.22 × 10^–19^) (Supplementary Data 4c).

### Sensitivity analyses

We undertook a series of sensitivity analyses to investigate the causal effect between height and CAD by instrumental variable analysis after sequentially excluding variants nominally associated with BMI, blood pressure (BP) or lipids (*p* < 0.05 were excluded). In each case the remaining variants constitute valid instruments and their causal effect estimates will therefore be immune to confounding. After excluding the variants associated with BMI, a 1 standard deviation increase in height, measured by the genetic score (weighted genetic score gave very similar results), was associated with 22% lower risk of CAD (OR = 0.77, 95% CI 0.73–0.81), the same as the observed effect without instrument exclusions. Similar results were obtained also after excluding variants associated with any lipid trait, a 1 standard deviation higher height was associated with a 13% decrease in the odds of CAD (OR = 0.83, 95% CI = 95% 0.78–0.88), whereas exclusion of variants associated with BP resulted in an 15% lower risk (OR = 0.85, 95% CI 0.76–0.94) of CAD (Supplementary Data 5, Supplementary Fig. 3).

Sensitivity analyses to investigate the causal effect of height on T2D (1 SD increase in height was associated with an OR of 0.90) resulted in an attenuation of the height effect after removing BMI, lipid or BP associated variants from the two-stage analysis (Supplementary Data 5, Supplementary Fig. 3).

### Two-sample Mendelian randomisation analyses

To further investigate the causal relationships found using two-stage analysis in UKBB and also test the validity of the genetic score as an instrument, two-sample Mendelian randomisation approaches were used to detect and accommodate violations of the Mendelian randomisation assumptions, specifically horizontal pleiotropy.

We accessed summary statistics from the largest genetic studies publically available for height (up to 700,000 individuals), CAD (up to 71,000 cases), and T2D (up to 27,000 individuals). Two-sample Mendelian randomisation analyses were performed using the inverse-variance weighted (IVW) method^23^, alongside other methods to overcome the violations of specific instrumental variable assumptions, as no single method controls for all statistical properties that may impact Mendelian randomisation estimates, including: Inverse-variance-weighted; MR-Egger (Egger); weighted median, mode-based estimate (MBE)^24^, generalised summary-data-based Mendelian randomisation (GSMR)^25^ and MR-PRESSO^26^ approaches.

Consistency in results across methods builds confidence in the obtained estimates, as they rely in different assumptions and models of horizontal pleiotropy. Funnel plots were also assessed for any deviations which can be suggestive of pleiotropy. We note that the plots appear generally symmetrical, suggesting no evidence for horizontal pleiotropy (Supplementary Figs 4b, 5b).

**Fig. 3:**
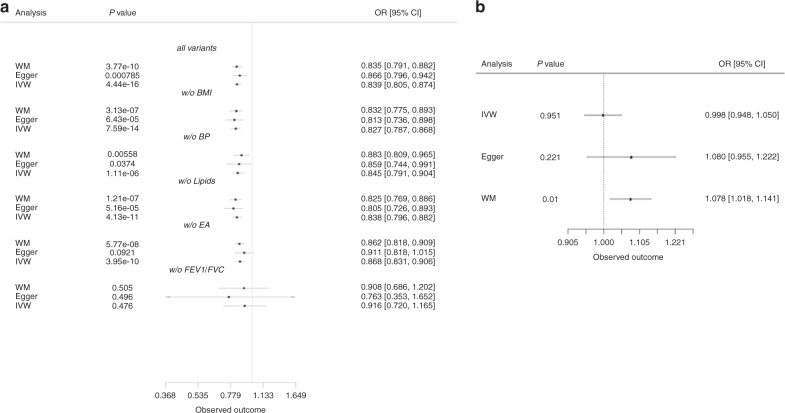
Two sample Mendelian randomisation analyses. Estimates of the effect of height on **a** coronary artery disease after removing variants nominally associated with BMI, lipids or blood pressure and **b** Type 2 diabetes adjusted for BMI. Effect estimates represent the ORs (95% CI)

IVW analysis indicated a causal effect of increased height lowering CAD risk (Fig. 3) consistent in direction with the instrumental variable analyses. There was little evidence of heterogeneity in the analysis (*p* = 0.9). The slope from the Egger regression was consistent with these findings (OR of 0.86 per 1 standard deviation higher height, 95% CI 0.79–0.94), and no evidence of directional pleiotropy (Intercept = −0.0009, 95% CI −0.0029 to 0.0012) (Fig. 3, Supplementary Figs 4, 5). We measured a low dilution bias in the MR-Egger casual effect, 97.5% through the *I*^2^ index of gene-exposure estimates, suggesting no violation of the NO measurement error assumption (NOME) assumption (see Methods) (Supplementary Data 6). The results obtained using the weighted median approach further confirmed the direction and magnitude of effect seen with the other methods (OR = 0.83, 95% CI 0.81–0.85), providing no evidence for pleiotropy (Supplementary Data 6, Fig. 3). The MBE method, which relaxes the instrumental variable assumptions and presents less bias and lower type-I error rates than the other methods, gave similar results; one standard deviation higher height was associated with 18% decrease in the odds of CAD with the weighted method assuming the NOME assumption is valid (OR = 0.82, 95% CI 0.73–0.92), setting the bandwidth tuning parameter φ equal to 1 (Supplementary Data 7). Finally, the GSMR method suggested that 1 standard deviation higher height was associated with 16% decrease in the risk of CAD (OR = 0.84, 95% CI 0.82–0.88, *p* = 2.91 × 10^−21^) (Supplementary Data 8), slightly higher than the estimate from a previous study^25^. MR-PRESSO results were in accordance with the other methods (Supplementary Data 8).

The results we obtained for CAD using two-sample Mendelian randomisation analyses were largely concordant with the two-stage analysis (Methods); 1 standard deviation increase in height (6.4 cm) was associated with a 14% (OR = 0.86) lower risk of CAD with no evidence for directional pleiotropy (Egger method) (Supplementary Data 6).

Sensitivity analyses showed that exclusion of variants associated with either BMI, BP, or lipid levels (i.e., one trait at a time) slightly increased the signal, a 1 standard deviation higher height (cm) was associated with a 19% (-BMI variants), 14% (-BP) or 19% (-lipids) lower risk of CAD (Supplementary Data 9–14, Fig. 3a). After exclusion of any height variant nominally associated with any of BMI, lipids or BP, the remaining 155 height associated variants yielded the strongest effect with 1 standard deviation increase in height associated with 21% lower risk of CAD (OR = 0.79, 95% CI 0.64–0.97, *p* = 0.02; Supplementary Data 15). Exclusion of variants nominally associated with age completed full time education didn’t affect initial estimates for CAD (OR = 0.87, 95% CI 0.83–0.90, *p* = 3.95 × 10^−10^; Supplementary Data 16). Removing variants associated with lung function measure by FEV1 and FVC completely abolished the effect (OR = 0.92, 95% CI 0.71–1.17, *p* = 0.476; Supplementary Data 17). The IVW method indicated a causal association between height and T2D (OR = 0.93, 95% CI 0.89–0.98) and there was no evidence of directional pleiotropy (Intercept = −0.00206, 95% CI −0.00529 to 0.0011) (Supplementary Data 18, Fig. 3b, Supplementary Fig. 5). Exclusion of variants associated with BMI resulted in a nominally significant causal effect of height on T2D risk (*p* = 0.04), but not when we excluded variants associated with lipids (*p* = 0.14) or BP (*p* = 0.08) (Supplementary Data 19–22, Fig. 3b).Consistent with our results from the two-stage analysis in UKBB, IVW analysis when T2D was adjusted for BMI showed no causal effect of height on T2D (*p* = 0.95) (Supplementary Data 23). The mode-based, GSMR, MR PRESSO and IVW-MR assuming random effects methods were in accordance with the other methods we applied (Supplementary Data 6–8, 23, 24).

### Mediation analyses

As described above, BMI adjustment in the Mendelian randomisation analyses showed complete attenuation of the causal effect of height on T2D and a modest decrease of the effect on CAD. Also, when we performed sensitivity analyses i.e., by excluding all BMI associated variants the results were unchanged. So, the Mendelian randomisation assumption of no correlation with potential confounders (i.e., BMI) was fulfilled. Therefore, we explored the role of BMI in the relation between height and CAD or T2D. Valid instruments for height and BMI were included. We applied a multiple-stage approach (see Methods) in UKBB to assess the direct genetic effect of height on CAD and T2D (Supplementary Data 25a). The causal effect of height on CAD after adjustment for genetic BMI was still significant (OR = 0.75, 95% CI 0.70–0.81, *p* = 7.32 × 10^−13^). However, for T2D no causal effect was observed in the multiple stage least square approach after adjustment for BMI (OR = 0.99, 95% CI 0.93–1.06, *p* = 0.835) (Supplementary Data 25a). In addition, we performed sensitivity analyses in order to investigate the robustness of the estimation in the above mediation analysis, by excluding any height associated variants which had a nominal association with BMI. This exclusion did not affect the previous observations (CAD: OR = 0.82, 95% CI 0.77–0.87, *p* = 2.53 × 10^−10^, T2D: OR = 0.98, 95% CI 0.92–1.03, *p* = 0.374) (Supplementary Data 25b).

**Fig. 4:**
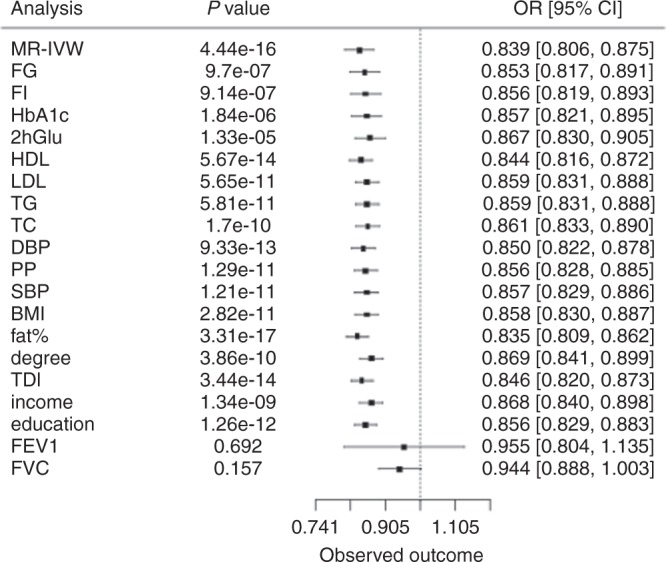
Multivariable separate-sample Mendelian randomisation analysis of the effect of height (per standard deviation) on CAD risk. MR-IVW: Mendelian randomisation inverse variance weighted; FG, free glucose; FI, free insulin; HbA1c, glycated haemoglobin; 2hGlu, Glucose 2 h tolerance test; HDL, High Density Lipoprotein; LDL, Low Density Lipoprotein; TG, triglycerides; TC, total cholesterol; DBP, diastolic blood pressure; PP, pulse pressure; SBP, systolic blood pressure; BMI, body mass index; fat%, body fat percentage; degree, College or University degree; TDI: Townsend deprivation index (a composite measure of deprivation based on unemployment, non-car ownership, non-home ownership and household overcrowding); income, income variable representing annual household income before tax; education, age in years at completion of full time education; FEV1, forced expiratory volume in 1 s; FVC, forced vital capacity

To increase power, we further investigated all the above mediation effects by multivariable Mendelian randomisation analysis (see Methods) using summary statistics data, in order to interrogate whether the exposure is causally associated with the outcome given the risk factors. Using this approach, we estimated the effect of height on CAD and T2D risk adjusting for the effect of each instrument with genetic BMI^27^. Similar analyses were performed for lipid levels, BP, glycaemic traits, lung function and socio-economic status. None of these factors, was found to be a strong mediator of the causal effect of height on the risk of CAD, association signal was attenuated by 1–3% in terms of magnitude. For example, the height-CAD effect reduced from 0.83 (95% CI 0.80–0.87) to 0.86 (95% CI 0.83–0.89) with adjustment for LDL (Supplementary Data 26, and 27, Fig. 4). In contrast, adjustment for FEV1 or FVC abolished the association suggesting that lung function acts as a mediator in the effect between height and CAD (Supplementary Data 27, Fig. 4). The causal effect of height on T2D was abolished after adjusting for the genetic effect of BMI, 2hGlu, lung function and socio-economic status variables (Supplementary Data 27b). For T2D adjusted for BMI, there was no evidence of a direct effect of height (OR = 0.99, 95% CI 0.96, 1.02, *p* = 0.95) (Supplementary Data 28, 27c, d). This finding did not change after taking into account the genetic effect of glycaemic, BP, lipid traits in multivariable Mendelian randomisation (Supplementary Data 27c, d). To further evaluate the robustness of the estimation in the mediation analyses, we performed the previous analyses by excluding variants nominally associated with BMI. The causal effect of height on T2D adjusted for BMI was also abolished, after excluding BMI associated variants (Supplementary Data 27d).

### Bidirectional Mendelian randomisation analysis

As a negative control, we investigated the effect of the known CAD associated variants^28^, T2D^29^ and lung function on adult height by two-sample Mendelian randomisation using summary statistics data.

The analysis using genetic variants related to CAD as instruments for height measurements, indicating no evidence for a causal effect of CAD (*p* = 0.57) on height (Supplementary Data 29).

When we performed the Mendelian randomisation analyses using variants related to T2D as instruments for height, there was no evidence of a causal effect of T2D on height (*p* = 0.39) and no evidence or directional pleiotropy (*p* = 0.62) from the MR-Egger regression (Supplementary Data 30). There was no evidence of a causal effect of lung function on height with the crude analysis (Supplementary Data 31).

## Discussion

In this study we investigated not only the causal relationship between adult height and cardiometabolic diseases (CAD and T2D) but also the extent to which traditional risk factors (obesity, glycaemic, lipid, and BP), lung function and socio-economic status may mediate such effects.

Consistent with previous studies^4,6,7,30^, our Mendelian randomisation results provided strong evidence for a protective causal effect of adult height on CAD risk, 1 standard deviation higher height (~6.5 cm) was causally associated with a 16% lower risk of CAD (OR = 0.84, 95% CI 0.80–0.87) using summary statistics data.

Our results suggest that the effect of height on CAD is not mediated via socio-economic status variables. Furthermore, this effect is not completely mediated by traditional cardiometabolic risk factors and may involve alternative biological pathways. For example, it has been postulated that the inverse association between height and CAD may be due to shorter individuals having higher BP^31^. Shorter individuals have increased heart rate, increased augmentation of the systolic pulse, which may increase ventricular systolic work^32^. Furthermore, shorter individuals have smaller vessel calibre, so their arteries can become more easily occluded and subsequently increase CAD risk^32–35^. However, exclusion of variants that are nominally associated with BP only marginally attenuated the causal association between height and CAD. Lipids appeared to have an even more modest effect in comparison to BP as possible mediators of the causal effect of adult height on CAD risk. There is also evidence suggesting that increased height is associated with increased risk of cancer and among mechanisms linking height with CAD and cancer are insulin and insulin-like growth factor signalling pathways^36^.

In contrast to the modest effects of BP and lipids as possible mediators of the effect of height on CAD, we found that lung function (as measured by FVC and FEV1) is a mediator. Mediation analyses accounting for the genetic effect of FEV1 or FVC abolished the association between height and CAD.

We did not find any evidence supportive of a direct causal effect of height on T2D, despite the modest association we observed based on observational data. Our results suggest that while there is an indirect effect on T2D, this effect is not direct and may be mediated by multiple factors (BMI, socio-economic status etc.).

A potential limitation of our study is that we have assumed no interaction between height and the mediators. However, we were unable to test for this as we used aggregated genome-wide data for glycaemic and lipid traits (unavailable in UK Biobank at the time of analyses). Also, selection bias is an issue when using any general population cohort, including the UK Biobank^37^. Such participants tend to be slightly healthier than the underlying population participants selected from. While UK Biobank participants are not representative of the general population (and hence cannot be used to provide representative disease prevalence and incidence rates), valid assessment of exposure-disease relationships are nonetheless widely generalisable and do not require participants to be representative of the population at large. In the two-sample Mendelian randomisation, where independent samples were used, weak instrument bias may result in bias towards the null. Similarly, in mediation analyses weak instrument bias could result in underestimation of the mediating effects. However, we assumed that estimates come from two different homogenous population studies without overlapping samples. The use of large sample sizes and instruments with large *F* statistics in our analyses is likely to have minimised any effect on the obtained results. Also, the IVW method has been shown to lead to slightly biased estimates (10% in either direction) in the presence of binary outcome and to a natural correlation between causal estimates and standard errors that could contribute to the presence of heterogeneity misinterpretable as pleiotropy^38^.

Adult height is known to be associated to different socio-economic factors^19,30^. Although we considered several socio-economic factors, it remains possible that we are not fully accounting for all confounding by other socio-economic status parameters in our Mendelian randomisation analyses^20^.

In summary, we show that the main mediator of causal effect of height on CAD is lung function whereas traditional CAD risk factors have only marginal effects. We also show that there is no evidence of a direct causal effect between height and T2D.

## Methods

### Analyses using individual level data

The UKBB recruited more than 500,000 individuals aged 37–73 between 2006 and 2010 across Great Britain. All participants provided information with questionnaires and interviews regarding health status, anthropometric characteristics as well as blood, urine and saliva samples^22^. Data underwent central quality control (see (http://biobank.ctsu.ox.ac.uk). UKBB samples were excluded due to sample relatedness determined as kinship coefficient greater than 0.0884.

### Continuous traits

Height (cm) was measured using a Seca 202 device in all participants of UKBB. We excluded individuals who exceeded *a* ± 5 standard deviation away from the mean of the sampled population.

BMI was constructed from height and weight measured during the initial Assessment Centre visit. Value is not present if either of these readings were omitted. Continuous traits were adjusted for demographics, genetic structure and converted to a normal distribution to limit the influence of any population stratification and provide standard deviation effect sizes. Residuals of the exposure from standard linear regression were taken by using as covariates: age, sex, five principal components and batch. The residuals were then inverse normalised in order to improve comparability with summary data Mendelian randomisation analysis.

### Disease definitions

CAD definitions: UKBB self-reported data: ‘Vascular/heart problems diagnosed by doctor' or ‘Non-cancer illnesses that self-reported as angina or heart attack’. Self-reported surgery defined as either PTCA, CABG or triple heart bypass. HESIN hospital episodes data and death registry data using diagnosis and operation—primary and secondary cause: MI defined as hospital admission or cause of death due to ICD9 410–412, ICD10 I21-I24, I25.2; PTCA is defined as hospital admission for PTCA (OPCS-4 K49, K50.1, K75); CABG is defined as hospital admission for CABG (OPCS-4 K40–K46); Angina or chronic IHD defined as hospital admission or death due to ICD9 413, 414.0, 414.8, 414.9, ICD10 I20, I25.1, I25.5–I25.9.

Type 2 diabetes definitions: UKBB self-reported data: ‘Diabetes by Doctor’ or “Non-cancer illnesses that self-reported as T2D’. HESIN hospital episodes data and death registry data using diagnosis and operation—primary and secondary cause: T2D defined as hospital admission or cause of death due to ICD10 E11.

Hypercholesterolaemia definitions: UKBB self-reported data: ‘Non-cancer illnesses that self-reported as Hypercholesterolaemia. HESIN hospital episodes data and death registry data using diagnosis and operation—primary and secondary cause: Hypercholesterolaemia defined as hospital admission or cause of death due to ICD10 E780, E7800, E7801.

### Observational associations

Whether observational associations between height and cardiometabolic disease have been documented, for consistency purposes we performed conventional regression analysis of each cardiometabolic disease (CAD and T2D) against height by using logistic regression. Height was the independent variable for each trait of interest by using linear and logistic regression for continuous and binary traits, respectively. These associations were adjusted for age, sex, first 40 PCs and batch. This information was compared with the estimates derived from instrumental variable analyses.

### Genetic analyses

Genotypes were extracted from UKBB imputation dataset (Supplementary Data 35: Summary of the height variants previously identified as associated with height at genome wide significance). Individual genotypes were excluded if the imputation quality was less than 0.4. We confirmed that the variants were imputed with high quality by comparing them with the directly genotyped data, where available. Eight hundred and twenty eight independent (*r*^2^ ≤ 0.05) and GWA significant (*p* < 5 × 10^−8^) SNPs were selected from the large GWA studies for height^13,14^.

### Genetic scores

Two genetic scores, weighted and unweighted, were created. The first incorporated 828 independent height associated variants. We pruned variants which were in linkage disequilibrium (LD) *r*^2^ of 0.05 Variants with low imputation quality or unavailable were excluded. Individual variants were recoded as 0, 1 and 2, depending on the number of height increasing alleles. These variants were used to create genetic scores.

The unweighted genetic scores for each individual were created by summing the number of height increasing alleles for the 828 SNPs they are carrying. Weighted genetic scores were also modelled. The weighted genetic score was calculated as the sum of the number of height-associated alleles, weighted by the relative effect size (β-coefficient) reported from the discovery meta-analysis^13,14^. In the derived weighted genetic score, β represents the association between an additional weighted height-associated allele at each single nucleotide polymorphisms (SNP) and height from large GWA meta-analyses^13,14^: weighted score = *β*_1_ × SNP_1_ + *β*_2_ × SNP_2_ + …*β*_*n*_ × SNP_*n*_. We present the range of the possible number of weighted height-increasing alleles, by dividing the score by the average effect size of the variants for each individual^39^. This is a transformation of the wGRS so that the range equalled that of the unweighted score. Linear regression for each score with height and logistic regression for each score with disease status were performed.

### Mendelian randomisation

SNPs from large GWAS study of height to date were identified by the 2015 and 2017 summary statistics files from the GIANT (Genetic Investigation of Anthropometric Traits) consortium. Data on effect and other alleles for each of the 828 LD pruned variants in up to 700,000 individuals of European descent, along with allele frequencies, beta coefficients for allele dose, and a 6.4 cm change in height, *p*-value and standard errors were extracted. In order to test the statistical significance of the association of the instrument with height, an *F* statistic was calculated using the formula: (β exposure × β exposure)/(se exposure × se exposure)^40^.

One condition of Mendelian randomisation is that exposure-related SNPs (the instrumental variables) must not be in LD with each other, as that can result in confounding^3^. For this purpose LD between all variants was estimated in European samples from 1000 Genomes using Plink software version 1.9^41^. When two or more SNPs were in LD (*r*^2^ > 0.05) only the most strongly associated variant with height, based on *p*-value, was kept.

Mendelian randomisation relies on certain assumptions (Supplementary Fig. 6). The instrumental variable is robustly associated with the exposure of interest. This can be evaluated by estimating the *F* statistic and the *R*^2^ value. It is substantial to have large studies, especially in instances where the instrumental variable explains a small amount of the variance in the exposure (*R*^2^). Genome wide association studies for height have yielded a large number of genetic variants that account for around 30% of height heritability. That allows the use of strong instruments to be developed. The instrumental variable has to be independent of any confounder^42–45^. When using individual level data, known confounders can be checked. In two-sample Mendelian randomisation, confounders can obstruct testing of this assumption due to lack of summary data results on the association between the candidate genetic instruments and the confounders. The instrumental variable is independent of the outcome, given the exposure and any possible confounders. The instrumental variable should not influence the outcome on an alternative path, other than through the exposure. This assumption is violated by horizontal pleiotropy, in which there are alternative pathways that the instrumental variable can affect the outcome.

We first performed an instrumental variable analysis (two-stage analysis) in UKBB, where we had access to individual level data, and then expanded this analysis to the largest summary statistics data sets currently available, assuming homogeneity among the studies. We used summary data from genetic studies of the associations of height associated variants with height from GIANT meta-analyses^13,14^. The associations of the height variants with the other traits were extracted from the following sets: CAD (CARDIoGRAMplusC4D-http://www.cardiogramplusc4d.org/data-downloads/), T2D (DIAGRAM)^29^ using two-sample Mendelian randomisation methods. To investigate potential mediators, genetic associations with fasting insulin, fasting glucose, 2hGlu and HbA1c were obtained from MAGIC, http://www.magicinvestigators.org/); HDL-cholesterol, LDL-cholesterol, total cholesterol and triglycerides were obtained from GLGC (http://csg.sph.umich.edu/abecasis/public/lipids2013/); anthropometric traits for GIANT (https://portals.broadinstitute.org/collaboration/giant/index.php/GIANT_consortium_data_files); and BP from ICBP^46^. Associations for lung function (FEV1 and FVC), socio-economic status variables and body fat percentage summary data were extracted from http://www.nealelab.is/uk-biobank. Summary statistics are provided in Supplementary Data 32–37.

### Instrumental variable analysis (two-stage analysis)

The Mendelian randomisation approach used in this study was based on the following assumptions: the height genetic scores had a strong association with measured height; the height genetic scores were not associated with confounding factors that could bias the observational association between height and cardiometabolic disease; the height score was related to the outcome only through its effect on the exposure, assuming a linear relationship between height and the logit-transformed outcome.

In order to estimate the causal effect of height on disease status we performed instrumental variable analysis by using height genetic score as instrument. For the binary traits, we used the two-stage estimator (logistic control function estimator)^47–49^. The analysis was performed in two stages. First, the association between height genetic score and height was assessed. These predicted values were then used as the independent variable and disease status as the dependent variable in a logistic regression model. Analyses were adjusted for age, sex, 40 principal components and batch effect.

### Two-sample Mendelian randomisation

Two-sample Mendelian randomisation was undertaken using genome-wide association summary data from separate samples, where data of the genotypes and the exposure of interest are available in one sample, and data on genotype and the outcome of interest are available in the other. For this part no ethical approval was sought as all data were derived from summary statistics of published GWAS studies, with no individual-level data used.

### Association of height variants with cardiometabolic traits

Coronary artery disease genotyping data were derived from the most recent meta-analysis of Cardiogram+C4D, which investigated the association of 7 M variants after imputation in up to 30,000 cases. The per-allele log-OR of CAD was extracted together with its standard error for each of the independent genome-wide significant height variants. Effect sizes were aligned to the height increasing allele.

The two-sample Mendelian randomisation was undertaken using previously described methods^50^. Wald ratios were estimated for each SNP by dividing the per allele log-OR for CAD (beta_gy) by the per-allele effect on height for each SNP (beta_gx). Standard error for each Wald ratio was derived from the standard error of the variant-outcome association divided by the variant-exposure association for each instrument. We calculated the Wald ratio estimate where outcome ~ genetic score and exposure ~ genetic score estimates were obtained using the previous regression models with the genetic score.

### Inverse-variance weighted (IVW) method

Conventional linear regression analysis of the variant-exposure association and variant-outcome association for each instrument was undertaken and weighted by inverse variance. The point estimate is equal to that derived from fixed-effect meta-analysis. The IVW method assumes that all variants are valid instrumental variables. An IVW corrected for the standard errors of each instrument method was also applied. In this approach we corrected for the correlation between the associations of the instrument with the exposure and the association of the instrument with the outcome. When the IVW method shows substantial heterogeneity, this means that there may exist alternative pathways through which the SNPs affect the outcome (horizontal pleiotropy). Heterogeneity for the Wald ratios was tested with the Cochran’s *Q* and quantified with the *I*^2^ index^51^. We also used the Mendelian Randomization package to perform IVW analysis assuming random effects^52^.

### MR-Egger method

MR-Egger method is more robust to potential violations of the standard instrumental variable assumptions. This method was used to address the issue of the aggregate unbalanced horizontal pleiotropy, which could violate the third assumption of instrumental variable analysis. MR-Egger is similar to the IVW method except that the intercept is not constrained to pass through the origin^50^.

MR-Egger method uses a weighted regression with an unconstrained intercept to regress the effect sizes of the variant-outcome associations against effect sizes of variant-exposure associations. The unconstrained intercept removes the assumption that all genetic variants are valid instruments. This method is less prone to confounding from possibly pleiotropic variants which could have stronger effects on outcomes compared to the effect on the exposure.

When a non-zero intercept from the MR-Egger is observed, that would suggest that there are pleiotropic effects; this could result in bias of the IVW estimates, which are in the direction indicated by the intercept term. The estimate for the effect of the exposure on the outcome, is provided by the slope of the MR-Egger. It is important to mention that this estimate is correct, taking into account an additional assumption, the InSIDE (instrument strength independent of direct effect) assumption, which means that the associations between the genetic variants and the exposure are independent of the effect that he variants have directly on the outcome. Funnel plots can be used to demonstrate the individual variant effects on the exposure and the outcome against the inverse of their standard error. When no pleiotropy is present, the instrumental variable estimates for each variant are symmetrically distributed around the point estimate. However, it is possible to understand the contribution of each instrumental variable on the overall *Q* statistic graphically.

IVW and Egger methods use weights that consider the SNP-exposure associations to be known rather than estimated. This is called as the NO measurement error assumption (NOME). We use an adaptation of the I^2^ statistic in order to quantify the strength of NOME violation for MR-Egger. This measure is called I_GX_^2^ and lies between 0 and 1. A high value of I_GX_^2^ and close to 1, indicates that dilution does not affect the standard MR-Egger analyses performed^51,53^. For the main analyses we used the first order weights, which correspond to the first term of the Taylor series expansion, which approximate the standard error of the Wald ratio estimate^53^. The IVW and the MR-Egger regression analyses were repeated using the second order weights, which correspond to the first two terms of the Taylor series expansion^51,54^.

### Weighted median method

The weighted median method was used to investigate pleiotropy^55^. In this method, the Mendelian randomisation estimates are ordered and weighted by the inverse of their variance. When more than 50% of the total weight comes from SNPs without pleiotropic effects, the median Mendelian randomisation estimate should remain unbiased. This method improves precision and is more robust to violations of the InSIDE assumption. If InSIDE holds, then MR-Egger is consistent, while the weighted median will be only if 50% of the total weight comes from SNPs without pleiotropic effects.

### Mode-based estimate (MBE)

The MBE method has presented less bias and type-I error rates in simulations than other methods under the null in many situations. The MBE relaxes the instrumental variable assumptions and is less prone to bias due to violations of the InSIDE assumption^24^.

### GSMR method

Given that the correlations between the SNP instruments could lead to biased (smaller) Mendelian randomisation standard errors^23^, we also applied the method Generalised Summary-data-based Mendelian randomisation (GSMR) that performs a multi-SNP Mendelian randomisation analysis using GWAS summary-level data accounting for the sampling variance in the estimated SNP effects and remaining LD between SNPs. The GSMR R-package implements the GSMR method to test for putative causal association between a risk factor and a disease. We used 1000 Genomes (European population) imputed data to create the LD correlation matrix^25^.

### MR-PRESSO

MR-PRESSO (Mendelian randomisation pleiotropy residual sum and outlier)  is a method that allows for the evaluation for horizontal pleiotropy in multi-instrument Mendelian randomisation analyses using genome-wide summary association data^26^.

### Power calculations

Calculations were performed using a non-centrality parameter-based approach^56^ implemented in the publically available tool mRnd (http://cnsgenomics.com/). Power calculations are provided in Supplementary Fig. 7.

### Sensitivity analyses

To further investigate the presence of pleiotropy and narrow down the set of height variants which may have a causal effect on the risk of CAD and T2D, we performed sensitivity analyses.

First, we assessed which variants may contribute to the total heterogeneity, by estimating the *Q* statistic for each instrument. We then used some different thresholds (5th (L1), 1st (L2), 0.19th (L3) percentile of a chi-squared with 1 degree of freedom) and excluded variants which had a *Q* > L3, *Q* > L2 and *Q* > L1.

Second, we assessed which variants are associated with any potential mediators including BMI, BP (SBP, DBP, pulse pressure) and lipids (LDL, HDL, TG, TC). We excluded any height associated variant which showed evidence for association with these traits.

### Mediation analyses

To estimate the effect of height on T2D and CAD taking into account the role of potential mediators, we performed multivariable Mendelian randomisation analyses, by using the IVW Mendelian randomisation method with summary statistics data, after adjusting for the effect of each instrument with the potential mediator^57^. We evaluated the proportion of the effect that is mediated by any of the potential mediators by the changes in the total effect of the genetically determined height and on the outcomes, assuming that the mediators are continuously measured variables (multivariable Mendelian randomisation). We estimated the total, direct and indirect effects of the risk factor on the outcome by using summary data^27^. It is recommended to provide estimates of the total and direct effects, but not the indirect effect, as calculation of the indirect effect relies on the linearity of the relationship that cannot occur with a binary outcome^27^.

Using individual level data we also applied a three stage approach where we first estimated the fitted values of the height genetic risk score with height, second the fitted values of a BMI genetic risk score with BMI and finally we used these fitted values to estimate the direct effect of height on the outcomes^49,58^. The weighted regression method for calculating the direct effect is also equivalent to a two-stage regression method, except that the first stage also regresses the mediator on the genetic variants, and the second stage regresses the outcome on fitted values of the exposure and fitted values of the mediator. This two-stage approach can be undertaken to estimate the direct effect when individual-level data are available^27,49^.

### Bidirectional Mendelian randomisation analyses

We performed bidirectional Mendelian randomisation analyses of the association of Coronary artery disease and height. To construct a genetic instrument for CAD, we used variants which reached genome-wide significance in the CARDIOGRAM+C4D consortium. For the SNP-exposure we used the effect estimates and standard error of the associations of each variant with CAD derived from the meta-analysis^28^. We used a similar approach for the bidirectional Mendelian randomisation analyses with T2D. We used genome-wide associated with T2D in the DIAGRAM consortium^29^. We also performed bidirectional Mendelian randomisation analysis by using 268 lung function associated variants (FVC, FEV1, FEV1/FVC) as exposure to interrogate the effect of lung function on height. Our results suggest that there is no evidence of a causal effect between lung function and height with the Egger regression. Two-sample Mendelian randomisation analyses were conducted as described above for height and coronary artery disease.

Statistical analysis was performed using R (version 3.4.3, the R Foundation for Statistical Computing, Vienna, Austria) software.

### Reporting summary

Further information on experimental design is available in the Nature Research Reporting Summary linked to this article.

## Supplementary information

Supplementary Information

Description of Additional Supplementary Files

Supplementary Data

Reporting Summary

## Data Availability

Individual level genetic and phenotypic data of UK Biobank participants are available at http://biobank.ctsu.ox.ac.uk. GWAS meta-analyses data for GIANT, CARDIOGRAM+C4D, DIAGRAM, GLGC, MAGIC, and ICBP were publically available and downloaded from the corresponding consortium sites. The authors declare that summary statistics data supporting the findings of this study are available within the paper and its supplementary information files.
