# Supplementary material for: Mendelian randomisation analyses find pulmonary factors mediate the effect of height on coronary artery disease
Source: Commun Biol. 2019 Mar 27;2:119. doi: 10.1038/s42003-019-0361-2 (PMC6437163; doi:10.1038/s42003-019-0361-2)
Supplement: Supplementary file 1 — Supplementary Information [file 42003_2019_361_MOESM1_ESM.pdf]

Supplementary Information

Supplementary Figures

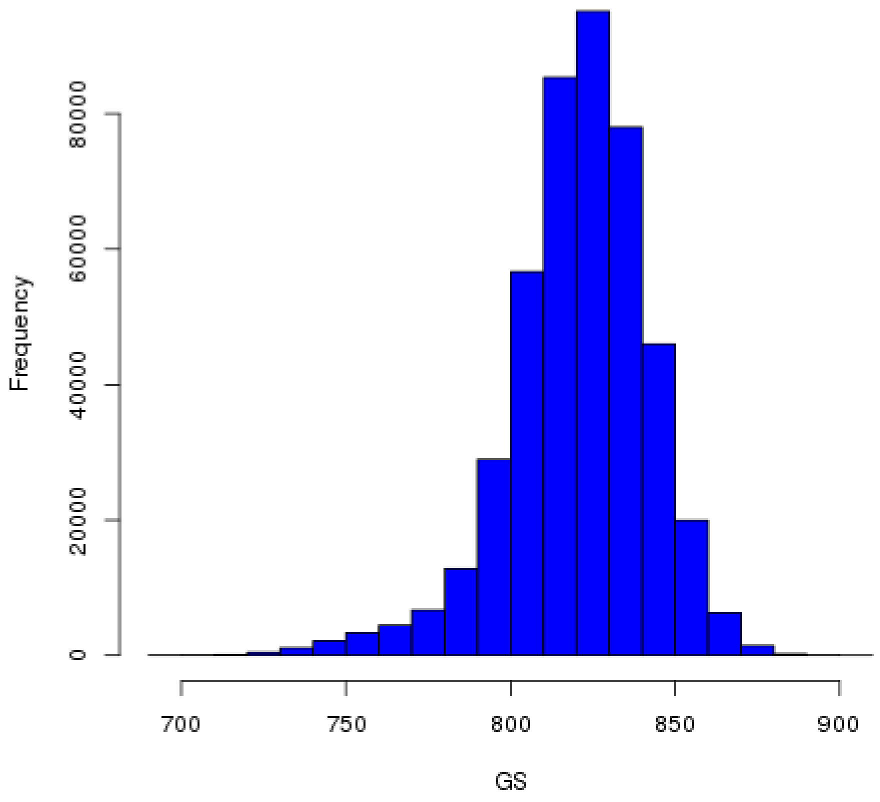

Supplementary Figure 1: Distribution of the unweighted height genetic score (GS) in UKBB

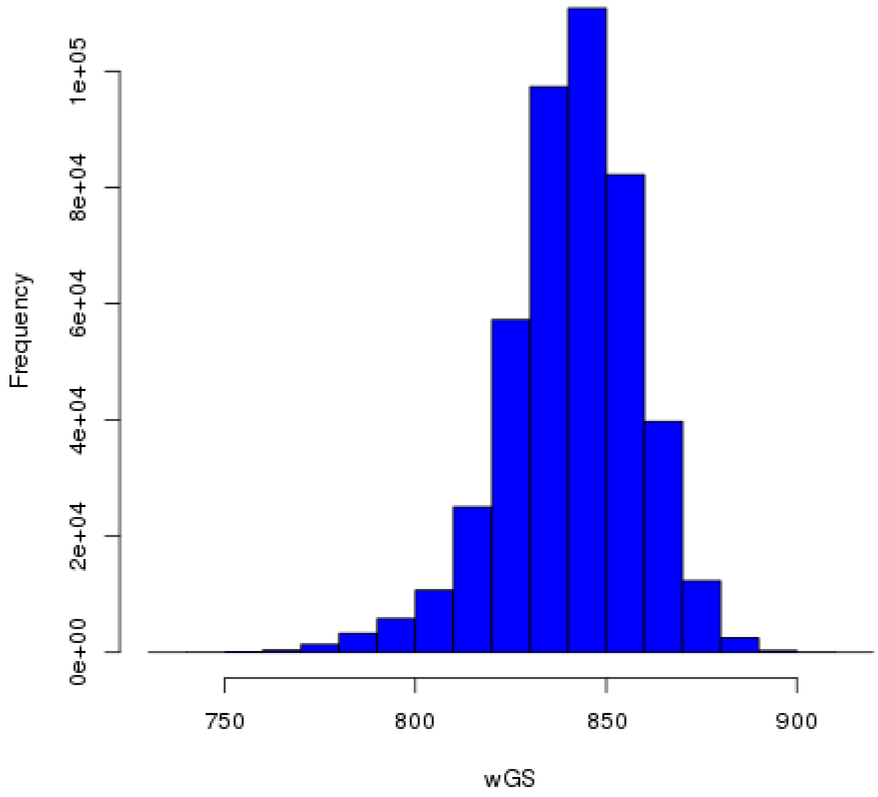

Supplementary Figure 2: Distribution of the weighted height genetic score (wGS) in UKBB

a.

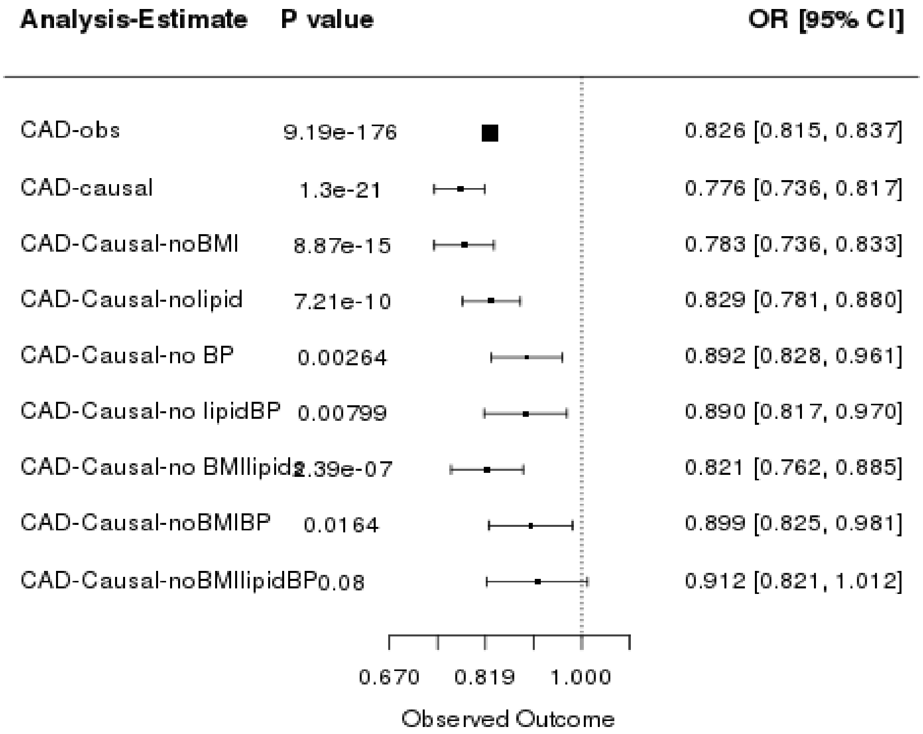
Analysis-Estimate
P value
OR [95% CI]
CAD-obs
9.19e-176
0.826 [0.815, 0.837]
CAD-causal
1.3e-21
0.776 [0.736, 0.817]
CAD-Causal-noBMI
8.87e-15
0.783 [0.736, 0.833]
CAD-Causal-nolipid
7.21e-10
0.829 [0.781, 0.880]
CAD-Causal-no BP
0.00264
0.892 [0.828, 0.961]
CAD-Causal-no lipidBP
0.00799
0.890 [0.817, 0.970]
CAD-Causal-no BMIlipid
2.39e-07
0.821 [0.762, 0.885]
CAD-Causal-noBMIBP
0.0164
0.899 [0.825, 0.981]
CAD-Causal-noBMIlipidBP
0.08
0.912 [0.821, 1.012]
0.670
0.819
1.000
Observed Outcome

b.

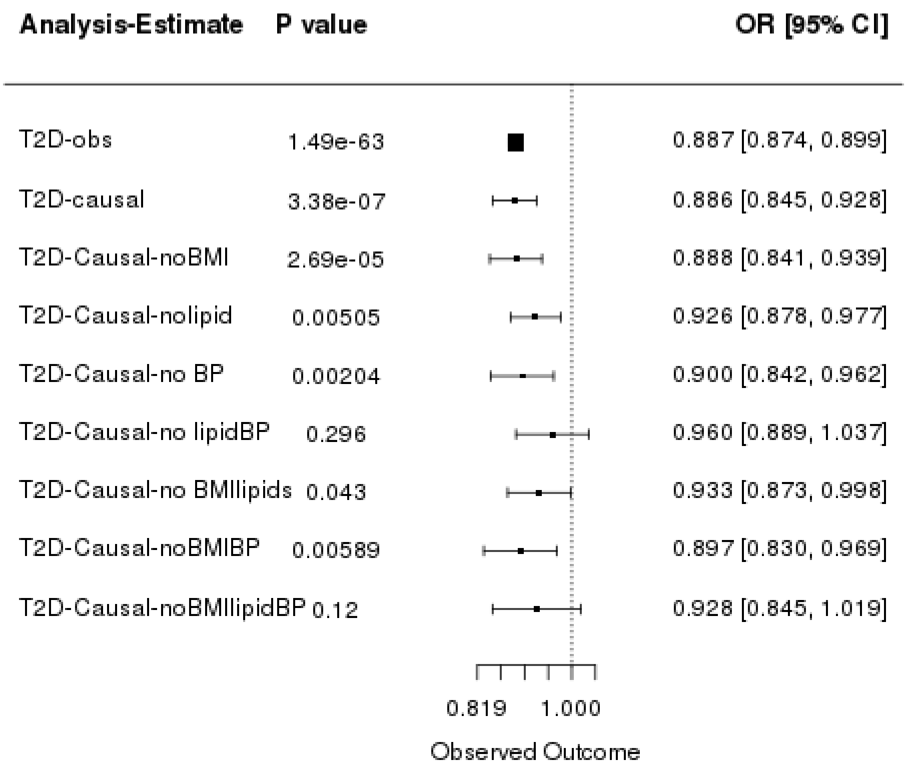

Supplementary Figure 3: Observational and Instrumental variable Estimates (using GS) of the Effect of height on Cardiometabolic disease status after removing variants nominally associated with BMI, lipids or blood pressure. a. CAD and b. Type 2 Diabetes.

Effect estimates represent the beta coefficients OR (95% CI)

a.

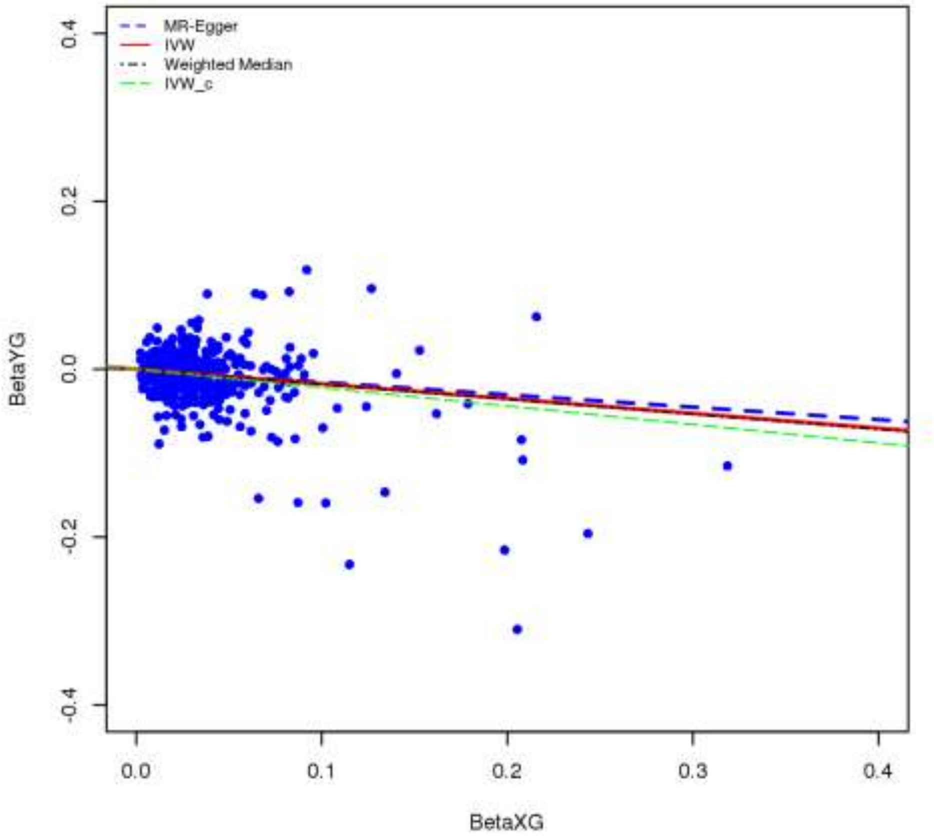

Supplementary Figure 4a: MR- Egger regression scatterplots for height on coronary artery disease. The red line shows the results of standard MR analysis (inverse-variance weighted (IVW), the green line shows the results from the IVW method with corrected SE, the black line the weighted median method (WM) and the blue line shows the pleiotropy adjusted MR-Egger regression line. The estimated slope of the MR-Egger regression, expressed as an OR, was 0.86 (95% CI= 0.79 to 0.94). The estimated MR-Egger intercept term was -0.001, 95% CI= -0.001 to 0012).

b.

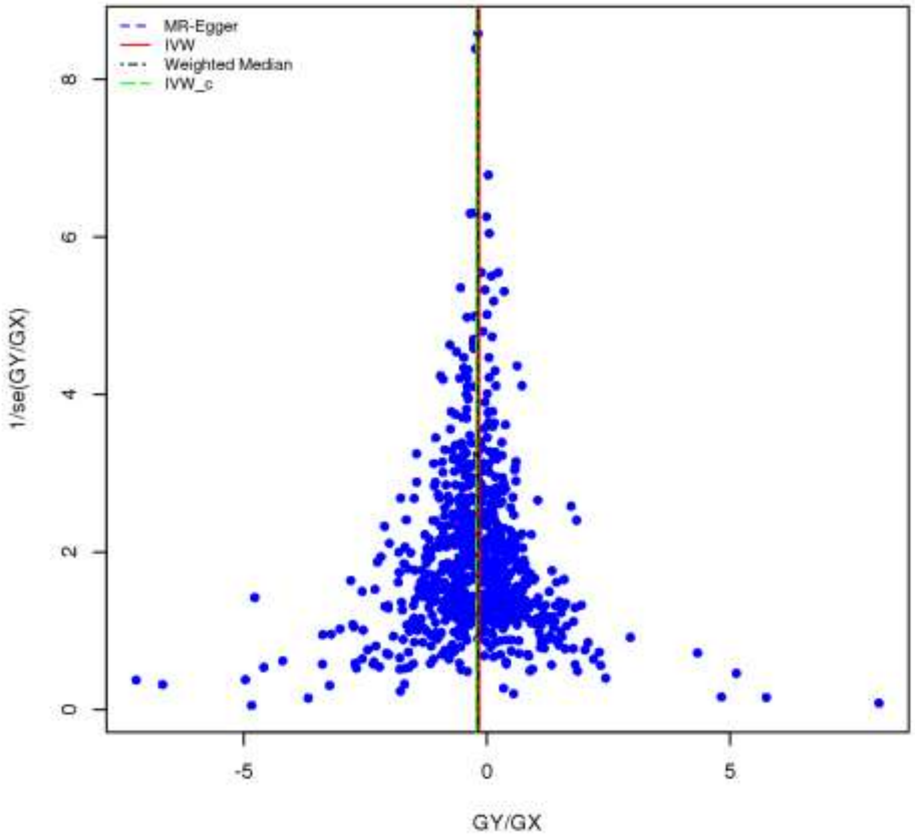

Supplementary Figure 4b: Funnel plot for height on Coronary Artery Disease. Each SNP 1/SE (GY/GX) is plotted against its Wald MR estimate (GY/GX). GY: association with outcome, GX: association with exposure. Similar to the use of funnel plots in the meta-analysis literature, this plot can be used for visual inspection of symmetry, where any deviations can be suggestive of pleiotropy. We note that the plot appears generally symmetrical.

a.

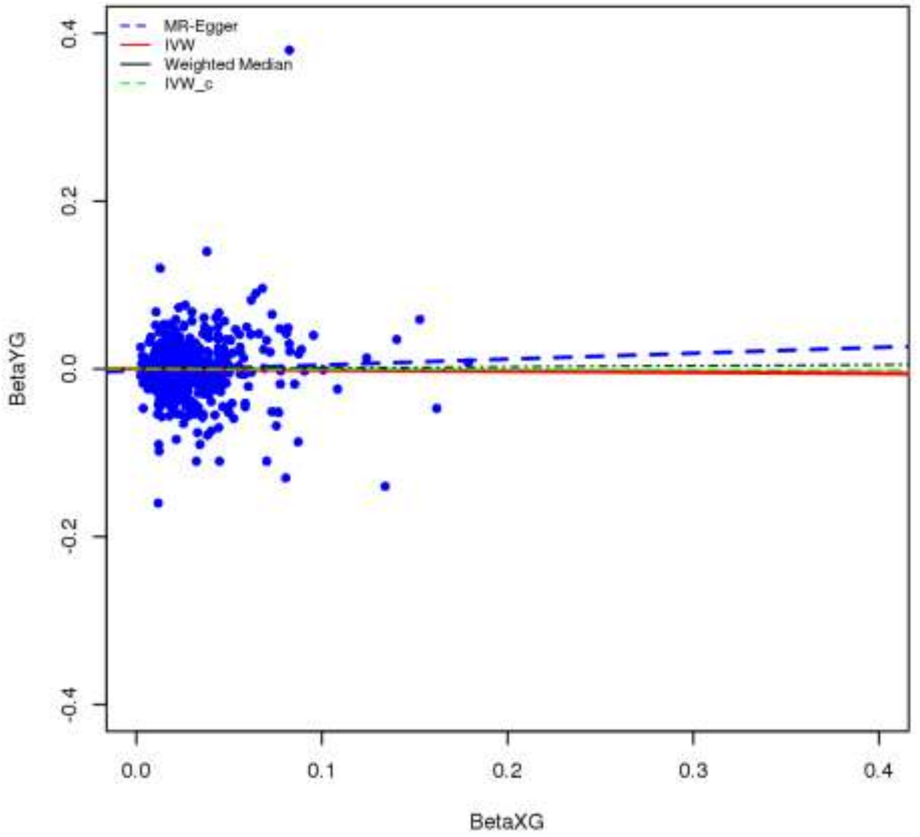

Supplementary Figure 5a: MR- Egger regression scatterplots for height on Type 2 Diabetes. The red line shows the results of standard MR analysis (inverse-variance weighted (IVW), the green line shows the results from the IVW method with corrected SE, the black line the weighted median method (WM) and the blue line shows the pleiotropy adjusted MR-Egger regression line. The estimated slope of the MR-Egger regression, expressed as an OR, was 0.86 (95% CI= 0.79 to 0.94). The estimated MR-Egger intercept term was -0.001, 95% CI= -0.001 to 0012).

b.

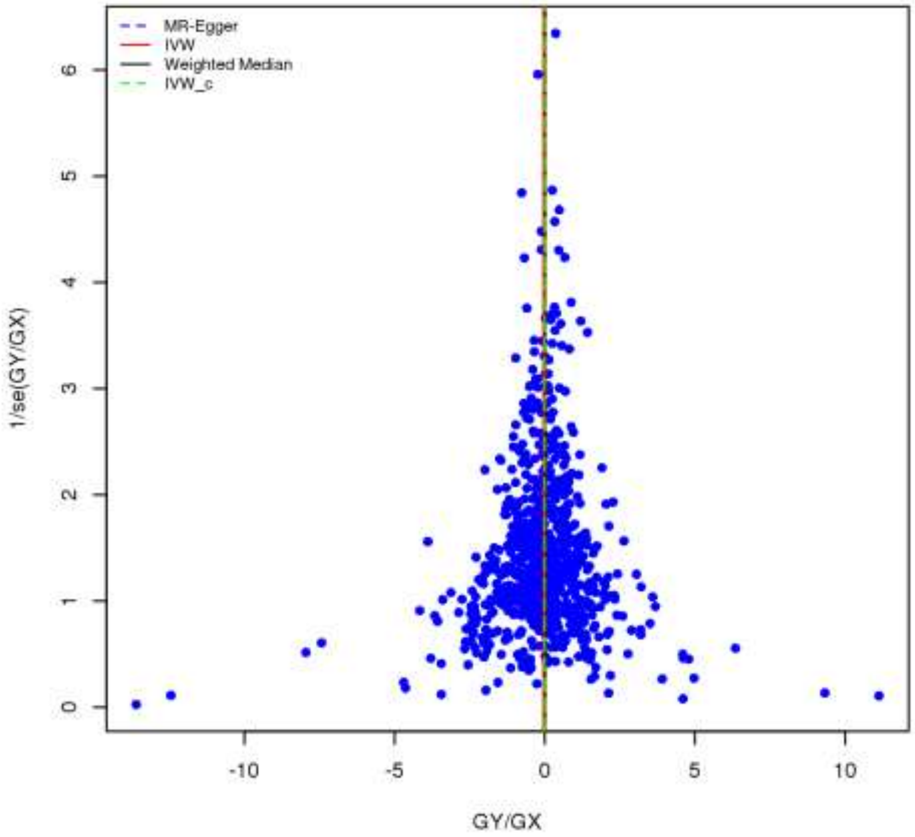

Supplementary Figure 5b: Funnel plot for height on Coronary Artery Disease. Each SNP 1/SE (GY/GX) is plotted against its Wald MR estimate (GY/GX). GY: association with outcome, GX: association with exposure. Similar to the use of funnel plots in the meta-analysis literature, this plot can

be used for visual inspection of symmetry, where any deviations can be suggestive of pleiotropy. We note that the plot appears generally symmetrical.

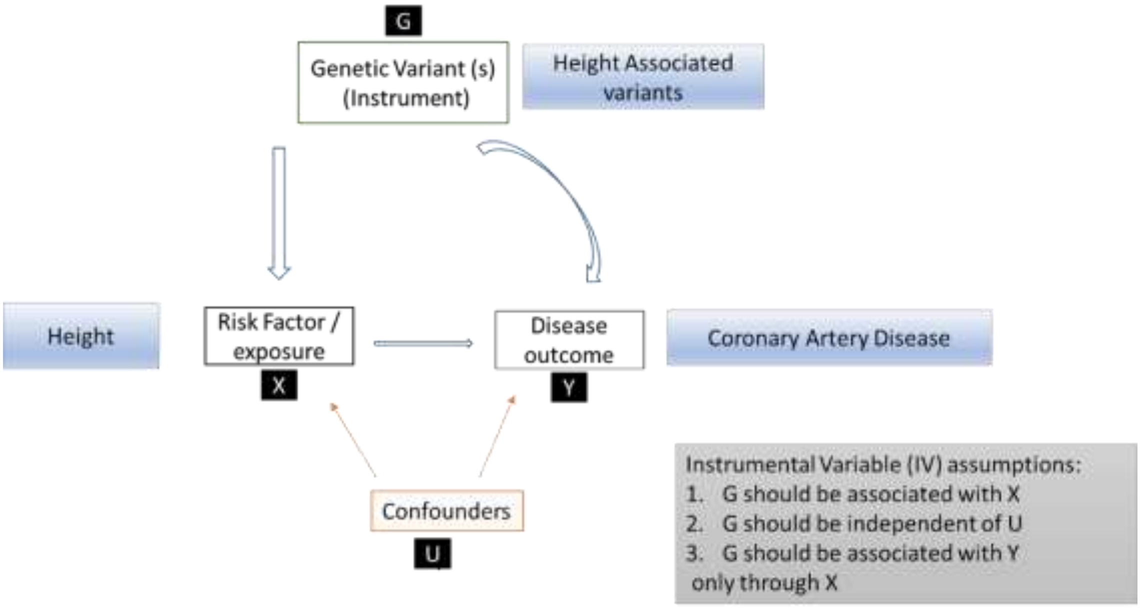

Supplementary Figure 6: Mendelian Randomisation Study Design

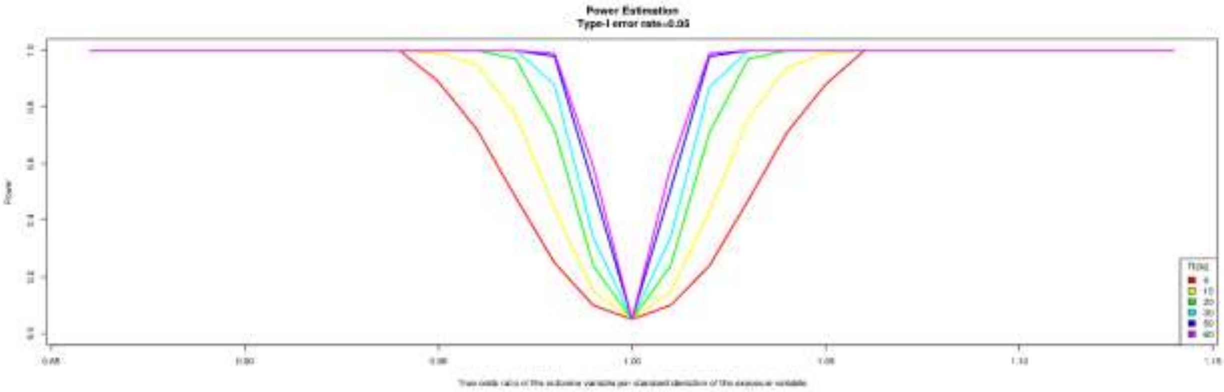

Supplementary Figure 7: Estimated power of the analyses for alpha: 0.05. Calculations were performed using mRnd (http://cnsgenomics.com/). Lines represent the different values of the proportion

of variance explained for the association between the SNP or allele score and the exposure variable (R2xz)
