# Supplementary material for: Mendelian randomisation analyses find pulmonary factors mediate the effect of height on coronary artery disease
Source: Commun Biol. 2019 Mar 27;2:119. doi: 10.1038/s42003-019-0361-2 (PMC6437163; doi:10.1038/s42003-019-0361-2)
Supplement: Supplementary file 2 — Description of Additional Supplementary Files [file 42003_2019_361_MOESM2_ESM.pdf]

Description of Additional Supplementary Data

File name: Supplementary Data 1
Description: Study-specific descriptive statistics of the cohort

File name: Supplementary Data 2
Description: Estimates of the Observational Relationship between Height and Cardiometabolic traits and events

Supplementary Data 2b. Estimates of the Observational Relationship between Height and Cardiometabolic events after adjustments for risk factors

File name: Supplementary Data 3
Description: Supplementary Data 3a. Estimates of the association between the height Genetic score and Cardiometabolic disease

Supplementary Data 3b. Estimates of the association between height Genetic score and Cardiometabolic events after adjustments for risk factors

File name: Supplementary Data 4
Description: Supplementary Data 4a.Two - stage analysis for Height and Cardiometabolic events

Supplementary Data 4b. Two stage - analysis for Height and Cardiometabolic events after adjusting for possible risk factors (BMI, BP, lipids).

File name: Supplementary Data 5
Description: Estimates of the Causal Relationship between Height and Cardiometabolic Disease after excluding variants associated with possible mediators (BMI, BP, lipids).

File name: Supplementary Data 6
Description: Two-sample Mendelian randomisation analysis of the effect of height on risk of CAD. OR=Odds ratio. Results are from the Inverse-variance weighted (IVW) method, the MR-Egger regression and the Weighted Median (WM) method. Q statistic (Q) for each instrument with thresholds (5th-L1, 1st-L2, 0.19th-L3 percentile of a chi-squared with 1 degree of freedom)

File name: Supplementary Data 7
Description: Two-sample Mendelian randomisation Analysis of the effect of height on risk of CAD with the 827 height-associated variants . Results are from the Mode-based estimate (MBE) approach.

File name: Supplementary Data 8

Description: Two- sample Mendelian randomisation analysis of the effect of height on risk of CAD and T2D with the height-associated variants. Results are from the GSMR (Generalised Summary-data-based Mendelian randomisation)and MR-PRESSO aproaches.

File name: Supplementary Data 9

Description: Two-sample Mendelian randomisation analysis of the effect of height on risk of CAD after removing variants nominally associatd with BMI. OR=Odds ratio. Results are from the Inverse-variance weighted (IVW) method, the MR- Egger regression and the Weighted Median (WM) method. Q statistic for each instrument with
thresholds (5th, 1st, 0.19th percentile of a chi-squared with 1 degree of freedom)

File name: Supplementary Data 10

Description: Two-sample Mendelian randomisation analysis of the effect of height on risk of CAD after removing variants nominally associatd with any Blood pressure Trait (SBP,DBP,PP). OR=Odds ratio. Results are from the Inverse-variance weighted (IVW) method, the MR-Egger regression and the Weighted Median (WM) method. Q statistic for each instrument with
thresholds (5th, 1st, 0.19th percentile of a chi-squared with 1 degree of freedom)

File name: Supplementary Data 11

Description: Two sample Mendelian randomisationAnalysis of the effect of height on risk of CAD after removing variants nominally associatd with any Lipid (LDL,HDL,TG,TC). OR=Odds ratio. Results are from the Inverse-variance weighted (IVW) method, the Egger regression and the Weighted Median (WM) method. Q statistic for each instrument with
threshols (5th, 1st, 0.19th percentile of a chi-squared with 1 degree of freedom)

File name: Supplementary Data 12

Description: Two sample Mendelian randomisationAnalysis of the effect of height on risk of CAD after removing variants nominally associatd with any Lipid (LDL,HDL,TG,TC) and any Blood Pressure trait (SBO, DBP, PP). OR=Odds ratio. Results are from the Inverse-variance weighted (IVW) method, the Egger regression and the Weighted Median (WM) method. Q statistic for each instrument with threshols (5th, 1st, 0.19th percentile of a chi-squared with 1 degree of freedom)

File name: Supplementary Data 13

Description: Two sample Mendelian randomisationAnalysis of the effect of height on risk of CAD after removing variants nominally associatd with any BP and BMI. OR=Odds ratio. Results are from the Inverse-variance weighted (IVW) method, the Egger regression and the Weighted Median (WM)

method. Q statistic for each instrument with
threshols (5th, 1st, 0.19th percentile of a chi-squared with 1 degree of freedom)

File name: Supplementary Data 14

Description: Two sample Mendelian randomisationAnalysis of the effect of height on risk of CAD after removing variants nominally associatd with any BMI and lipid. OR=Odds ratio. Results are from the Inverse-variance weighted (IVW) method, the Egger regression and the Weighted Median (WM) method. Q statistic for each instrument with
threshols (5th, 1st, 0.19th percentile of a chi-squared with 1 degree of freedom)

File name: Supplementary Data 15

Description: Two sample Mendelian randomisationAnalysis of the effect of height on risk of CAD after removing variants nominally associatd with any Lipid (LDL,HDL,TG,TC),any Blood Pressure trait (SBO, DBP, PP) and BMI. OR=Odds ratio. Results are from the Inverse-variance weighted (IVW) method, the Egger regression and the Weighted Median (WM) method. Q statistic for each instrument with
threshols (5th, 1st, 0.19th percentile of a chi-squared with 1 degree of freedom)

File name: Supplementary Data 16

Description: Two sample Mendelian randomisationAnalysis of the effect of height on risk of CAD after removing variants nominally associatd with age completed full time education in UK Biobank. Results are from the Inverse-variance weighted (IVW) method, the Egger regression and the Weighted Median (WM) method. Q statistic for each instrument with
threshols (5th, 1st, 0.19th percentile of a chi-squared with 1 degree of freedom)

File name: Supplementary Data 17

Description: Two sample Mendelian randomisation analysis of the effect of height on risk of CAD after removing variants nominally associatd with FEV1 and FVC in UK Biobank. Results are from the Inverse-variance weighted (IVW) method, the MR-Egger regression and the Weighted Median (WM) method. Q statistic for each instrument with
thresholds (5th, 1st, 0.19th percentile of a chi-squared with 1 degree of freedom)

File name: Supplementary Data 18

Description: Two sample Mendelian randomisation analysis of the effect of height on risk of T2D. OR=Odds ratio. Results are from the Inverse-variance weighted (IVW) method, the Mr-Egger regression and the Weighted Median (WM) method. Q statistic for each instrument with thresholds (5th, 1st, 0.19th percentile of a chi-squared with 1 degree of freedom)

File name: Supplementary Data 19
Description: Two sample Mendelian randomisationAnalysis of the effect of height on risk of T2D after removing variants nominally associatd with BMI. OR=Odds ratio. Results are from the Inverse-variance weighted (IVW) method, the Egger regression and the Weighted Median (WM) method. Q statistic for each instrument with
threshols (5th, 1st, 0.19th percentile of a chi-squared with 1 degree of freedom)

File name: Supplementary Data 20
Description: Two sample Mendelian randomisationAnalysis of the effect of height on risk of T2D after removing variants nominally associatd with BP traits. OR=Odds ratio. Results are from the Inverse-variance weighted (IVW) method, the Egger regression and the Weighted Median (WM) method. Q statistic for each instrument with
threshols (5th, 1st, 0.19th percentile of a chi-squared with 1 degree of freedom)

File name: Supplementary Data 21
Description: Two sample Mendelian randomisationAnalysis of the effect of height on risk of T2D after removing variants nominally associatd with lipid traits. OR=Odds ratio. Results are from the Inverse-variance weighted (IVW) method, the Egger regression and the Weighted Median (WM) method. Q statistic for each instrument with
threshols (5th, 1st, 0.19th percentile of a chi-squared with 1 degree of freedom)

File name: Supplementary Data 22
Description: Two sample Mendelian randomisationAnalysis of the effect of height on risk of T2D after removing variants nominally associatd with any Lipid (LDL,HDL,TG,TC),any Blood Pressure trait (SBO, DBP, PP) and BMI. OR=Odds ratio. Results are from the Inverse-variance weighted (IVW) method, the Egger regression and the Weighted Median (WM) method. Q statistic for each instrument with
threshols (5th, 1st, 0.19th percentile of a chi-squared with 1 degree of freedom)

File name: Supplementary Data 23
Description: Two sample Mendelian randomisationAnalysis of the effect of height on risk of T2Dadjusted for BMI. OR=Odds ratio. Results are from the Inverse-variance weighted (IVW) method, the Egger regression and the Weighted Median (WM) method. Q statistic for each instrument with threshols (5th, 1st, 0.19th percentile of a chi-squared with 1 degree of freedom)

File name: Supplementary Data 24
Description: Two sample Mendelian randomisation Analysis of the effect of height on risk of T2D with the 827 height-associated variants. Results are from the Mode-based estimate (MBE) approach.

File name: Supplementary Data 25
Description: Supplementary Data 25a: Results from the mediation analysis for the effect of height on CAD and T2D (multiple stage least square approach), using BMI as mediator in UK Biobank

Supplementary Data 25b: Results from the mediation analysis for the effect of height on CAD and T2D (multiple stage least square approach), using BMI as ediator in UK Biobank, after excluding variants nominally associated with BMI

File name: Supplementary Data 26
Description: Results from the mediation analysis for the direct and total effect of height on CAD.

File name: Supplementary Data 27
Description: Multivariate MR analysis of the effetc of height (per SD) on cardiometabolic traits (CAD and T2D)

File name: Supplementary Data 28
Description: Results from the mediation analysis for the direct and total effect of height on T2D adj BMI.

File name: Supplementary Data 29
Description: Bidirectional Mendelian randomisation Analysis of the association of CAD and height. OR=Odds ratio. Results are from the Inverse-variance weighted (IVW) method, the Egger regression and the Weighted Median (WM) method. Q statistic for each instrument with hreshols (5th, 1st, 0.19th percentile of a chi-squared with 1 degree of freedom)

File name: Supplementary Data 30
Description: Bidirectional Mendelian randomisation Analysis of the association of T2D and height. OR=Odds ratio. Results are from the Inverse-variance weighted (IVW) method, the Egger regression and the Weighted Median (WM) method. Q statistic for each instrument with hreshols (5th, 1st, 0.19th percentile of a chi-squared with 1 degree of freedom)

File name: Supplementary Data 31
Description: Mendelian randomisation Analysis of the association of lung function and height. OR=Odds ratio. Results are from the Inverse-variance weighted (IVW) method, the Egger regression and the Weighted Median (WM) method. Q statistic for each instrument with hreshols (5th, 1st, 0.19th percentile of a chi-squared with 1 degree of freedom)

File name: Supplementary Data 32
Description: Summary of the CAD associated variants and their association with CAD

File name: Supplementary Data 33
Description: Summary of the T2D associated and their association with T2D

File name: Supplementary Data 34
Description: Summary of the lung function associated variants and their association with pulmonary traits

File name: Supplementary Data 35
Description: List of the height variants previously identified from GIANT consortium as associated with height at genome wide significance

File name: Supplementary Data 36
Description: Summary of the height associated variants and their association with CAD

File name: Supplementary Data 37
Description: Summary of the height associated variants and their association with A. T2D and B. T2DadjBMI
