# Supplementary material for: Mendelian randomisation analyses find pulmonary factors mediate the effect of height on coronary artery disease
Source: Commun Biol. 2019 Mar 27;2:119. doi: 10.1038/s42003-019-0361-2 (PMC6437163; doi:10.1038/s42003-019-0361-2)
Supplement: Supplementary file 4 — Reporting Summary [file 42003_2019_361_MOESM4_ESM.pdf]

nature research

Corresponding author(s): Eirini Marouli

Last updated by author(s): Dec 12, 2018

Reporting Summary

Nature Research wishes to improve the reproducibility of the work that we publish. This form provides structure for consistency and transparency in reporting. For further information on Nature Research policies, see Authors & Referees and the Editorial Policy Checklist.

Statistics

For all statistical analyses, confirm that the following items are present in the figure legend, table legend, main text, or Methods section.

n/a	Confirmed	
☐	☒	The exact sample size (*n*) for each experimental group/condition, given as a discrete number and unit of measurement
☐	☒	A statement on whether measurements were taken from distinct samples or whether the same sample was measured repeatedly
☐	☒	The statistical test(s) used AND whether they are one- or two-sided<br>*Only common tests should be described solely by name; describe more complex techniques in the Methods section.*
☐	☒	A description of all covariates tested
☐	☒	A description of any assumptions or corrections, such as tests of normality and adjustment for multiple comparisons
☐	☒	A full description of the statistical parameters including central tendency (e.g. means) or other basic estimates (e.g. regression coefficient) AND variation (e.g. standard deviation) or associated estimates of uncertainty (e.g. confidence intervals)
☐	☒	For null hypothesis testing, the test statistic (e.g. *F*, *t*, *r*) with confidence intervals, effect sizes, degrees of freedom and *P* value noted<br>*Give P values as exact values whenever suitable.*
☒	☐	For Bayesian analysis, information on the choice of priors and Markov chain Monte Carlo settings
☒	☐	For hierarchical and complex designs, identification of the appropriate level for tests and full reporting of outcomes
☒	☐	Estimates of effect sizes (e.g. Cohen's *d*, Pearson's *r*), indicating how they were calculated

Our web collection on statistics for biologists contains articles on many of the points above.

Software and code

Policy information about availability of computer code

Data collection	No code used for data collection
Data analysis	Power calculations were performed using a non-centrality parameter-based approach implemented in the publically available tool mRnd (http://cnsgenomics.com/).<br>Statistical analysis was performed using R (version 3.4.3, the R Foundation for Statistical Computing, Vienna, Austria) software.

For manuscripts utilizing custom algorithms or software that are central to the research but not yet described in published literature, software must be made available to editors/reviewers. We strongly encourage code deposition in a community repository (e.g. GitHub). See the Nature Research guidelines for submitting code & software for further information.

Data

Policy information about availability of data

All manuscripts must include a data availability statement. This statement should provide the following information, where applicable:

- Accession codes, unique identifiers, or web links for publicly available datasets
- A list of figures that have associated raw data
- A description of any restrictions on data availability

Individual level genetic and phenotypic data of UK Biobank participants are available at http://biobank.ctsu.ox.ac.uk. GWAS meta-analyses data for GIANT, CARDIOGRAM+C4D, DIAGRAM, GLGC, MAGIC, and ICBP were publically available and downloaded from the corresponding consortium sites. The authors declare that summary statistics data supporting the findings of this study are available within the paper and its supplementary information files.

Field-specific reporting

Please select the one below that is the best fit for your research. If you are not sure, read the appropriate sections before making your selection.

☒ Life sciences ☐ Behavioural & social sciences ☐ Ecological, evolutionary & environmental sciences

For a reference copy of the document with all sections, see nature.com/documents/nr-reporting-summary-flat.pdf

Life sciences study design

All studies must disclose on these points even when the disclosure is negative.

Sample size	449,094 unrelated British participants from the UK Biobank. 23,755 individuals had CAD and 29,427 had T2D
Data exclusions	UKBB samples were excluded due to sample relatedness determined as kinship coefficient greater than 0.0884.<br>We excluded individuals who exceeded a +-5 SD height away from the mean of the sampled population.<br>Individual genotypes were excluded if the imputation quality was less than 0.4.
Replication	NA
Randomization	NA
Blinding	NA

Reporting for specific materials, systems and methods

We require information from authors about some types of materials, experimental systems and methods used in many studies. Here, indicate whether each material, system or method listed is relevant to your study. If you are not sure if a list item applies to your research, read the appropriate section before selecting a response.

Materials & experimental systems

n/a	Involved in the study	
☒	☐	Antibodies
☒	☐	Eukaryotic cell lines
☒	☐	Palaeontology
☒	☐	Animals and other organisms
☐	☒	Human research participants
☒	☐	Clinical data

Methods

n/a	Involved in the study	
☒	☐	ChIP-seq
☒	☐	Flow cytometry
☒	☐	MRI-based neuroimaging

Human research participants

Policy information about studies involving human research participants

Population characteristics	The UKBB recruited more than 500,000 individuals aged 37-73 between 2006 and 2010 across Great Britain.
Recruitment	See UKBB. All participants provided information with questionnaires and interviews regarding health status, anthropometric characteristics as well as blood, urine, and saliva samples. See https://www.nature.com/articles/s41586-018-0579-z for details.
Ethics oversight	Ethics approval for the UK Biobank study was obtained from the North West Centre for Research Ethics Committee (11/NW/0382).

Note that full information on the approval of the study protocol must also be provided in the manuscript.
